# The Treatment of Aquaculture Wastewater with Biological Aerated Filters: From the Treatment Process to the Microbial Mechanism

**DOI:** 10.3390/toxics11060478

**Published:** 2023-05-25

**Authors:** Jiafeng Ding, Yunjuan Meng, Shihuan Lu, Yiwen Peng, Wen Yan, Wenbing Li, Jinchun Hu, Ting Ye, Yuchi Zhong, Hangjun Zhang

**Affiliations:** 1 School of Life and Environmental Sciences, Hangzhou Normal University, Hangzhou 311121, Chinadjfphd@163.com (H.Z.); 2 School of Engineering, Hangzhou Normal University, Hangzhou 311121, China; 3 Zhe Jiang Sunda Public Environmental Protection Co., Ltd., Hangzhou 311000, China; 4 Quzhou Aquatic Technology Extension Station, Quzhou 324000, China

**Keywords:** biological aerated filters, aquaculture wastewater, algae cells, *M. polymorpha*, ammonia nitrogen

## Abstract

Algal cell proliferation has posed significant problems for traditional water treatment facilities; these problems are attributed to surface hydrophilicity and electrostatic repulsion. Biological aerated filters (BAFs) have been extensively used in wastewater treatment to remove pollutants such as algal cells by utilizing the adsorption and separation capabilities of the filter media. In this study, a BAF was supplemented with biological filter medium (*Marchantia polymorpha*) to assess its effectiveness of pretreating aquaculture wastewater. In terms of process performance, steady and consistent treatment was achieved by the BAF with *M. polymorpha* (BAF2) under an algal cell density as high as 1.65 × 10^8^ cell/L, with average removal rates for NH_4_^+^-N and algae cells of 74.4% and 81.9%, respectively. The photosynthetic activity parameters (rETRmax, α, Fv/Fm, and *I_k_*) of the influent and effluent were quantitatively assessed, and *M. polymorpha* was found to remove algae by disrupting the photosynthetic system of the algal cells. Furthermore, the addition of the *M. polymorpha* filter medium enhanced the community structure of the functional microbes in the BAF system. The highest microbial community richness and diversity were observed in the BAF2. Meanwhile, *M. polymorpha* promoted an increase in the abundance of denitrifying bacteria, including *Bdellovibrio* and *Pseudomonas*. Overall, this work offers a unique perspective on the aquaculture wastewater pretreatment process and BAF design.

## 1. Introduction

The random release of a massive volume of nitrogen- and phosphorus-rich aquaculture wastewater gives rise to the frequent occurrence of water eutrophication in the natural environment [1]. Aquaculture wastewater has also become a breeding ground for harmful algae [2]. Algae cells are difficult to separate from water due to electrostatic repulsion and surface hydrophilicity, which has caused severe challenges for conventional drinking water treatment facilities [3]. Ammonia nitrogen (NH_4_^+^-N) is a contaminant commonly detected in aquaculture wastewater [4,5]. Excessive concentrations of NH_4_^+^-N (>1.5 mg/L) can cause dissolved oxygen depletion and water quality decline and pose a threat to the survival of aquatic organisms [6]. Moreover, as coexisting pollutants, NH_4_^+^-N and algae cells may also react with chlorine during the chlorination disinfection to generate disinfection byproducts (DBPs), which cause harm to human health [7]. However, conventional drinking water treatment processes such as photocatalysis [8], coagulation–flocculation [9], and advanced oxidation processes [10] have difficulty removing algae cells and NH_4_^+^-N simultaneously and efficiently. Thus, the development of a pretreatment process for aquaculture wastewater is of great significance for maintaining drinking water safety.

Compared with conventional drinking water pretreatment utilities, biological aerated filters (BAFs) have overwhelming predominance owing to their compact footprint, large treatment volume capacity, and great resistance to shock loading [11]. BAFs can combine a short hydraulic retention time with a long sludge age organic unity which not only promotes the enrichment of nitrification bacteria with long generation periods but also obtains a high NH_4_^+^-N removal efficiency [12,13]. Therefore, BAFs have great potential for the treatment of aquaculture wastewater. In fact, the choice of filter medium has a significant impact on the treatment efficiency of a BAF. On one hand, suspended solids and micropollutants in the influent are removed by the filtration effect of the filter medium. On the other hand, microorganisms attached to the filter medium remove micropollutants and fine particles by flocculation. In addition, the multiplicity of filter medium will provide more possibilities to treat different wastewaters using BAFs [14]. Regarding textile wastewater, BAFs with zeolite media have advantages in removing color and organic matter [15]. Dong et al. [16] designed a high-performance BAF packed with porous ceramsites for the treatment of organic wastewater. Thus, the method of selecting appropriate filter medium for the effective pretreatment of aquaculture wastewater is of great research significance.

*Marchantia polymorpha* could be a potential filter medium for achieving a high removal efficiency of algal cells from aquaculture wastewater in BAF systems. *M. polymorpha* is a primordial terrestrial plant that releases secondary metabolites such as ethereal terpenoids and lipophilic aromatics, which can inhibit the growth of algal cells [17]. Thus, turning *M. polymorpha* into a biological resource to inhibit the growth of algal cells from aquaculture wastewater should be considered. *M. polymorpha* grows in a broad range of environments, making the material more accessible. Therefore, in response to the treatment requirements of aquaculture wastewater in this research, a unique BAF is set up, using *M. polymorpha* as the filler medium.

A conventional BAF was applied to remove pollution such as nitrogen and organic matter from wastewater via the activity of the microorganisms attached to the filler medium. To date, very few studies have reported the use of a natural filler medium in combination with a BAF to treat aquaculture wastewater. In this work, two aerating upflow biofilters were utilized to investigate the response of *M. polymorpha* to the removal efficiency of NH_4_^+^-N and algal cells. The difference between the two reactors was whether *M. polymorpha* was used. In parallel, the algae removal mechanism of *M. polymorpha* in aquaculture wastewater was revealed using batch experiments. With a focus on process performance, the dynamic changes in the microbial communities of the biofilms attached to *M. polymorpha* were also thoroughly plumbed in this study. The major purpose of this research was to uncover the underlying response mechanisms of *M. polymorpha* in BAF systems, as well as the role it plays in BAF operations, which would provide guidance for aquaculture wastewater pretreatment.

## 2. Materials and Methods

### 2.1. Characteristics of Aquaculture Wastewater and Seeding Sludge

The archetypical harmful cyanobacteria, *Microcystis aeruginosa* strain FACHB-905, was obtained from the Freshwater Algae Culture Collection of the Institute of Hydrobiology (Wuhan, China). A sterile BG-11 medium was used for culturing the test strain at 25 °C with light and dark periods (12/12 h) and 2000 lux illumination. Prior to the experiment, the test strain needed to reach exponential growth. Synthetic aquaculture wastewater was prepared following the recipe described in Table 1 and was used as the influent for the reactors. The biological aerated filter (BAF) was inoculated with 500 mL of activated sludge. The activated sludge was acquired from a denitrification reactor in our laboratory.

### 2.2. BAF Setup and Operation

**Figure 1:**
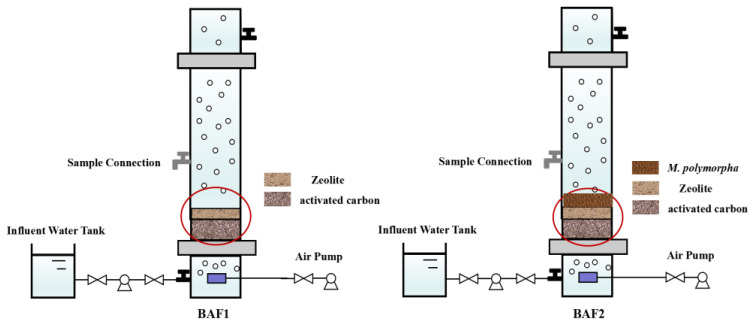
Schematic diagram of BAF1 and BAF2.

Two biological aerated filters named BAF1 and BAF2 were constructed in this study (Figure 1). All BAFs were composed of a transparent polyethylene plastic column and a PVC pipe. The column had a depth of 450 mm and a diameter of 150 mm, with an effective working capacity of 5 L. Both BAF1 and BAF2 contained zeolite and activated carbon as biomass carriers, and the filling height of both the zeolite and activated carbon was 50 mm. The gravel was 15~25 mm below the filter media as a support layer. The difference between BAF1 and BAF2 was the addition of a biological filler (*M. polymorpha*). BAF2 was utilized as an experimental group to investigate the influence of the addition of a biological filler on the nitrogen removal performance of the reactor, while BAF1 was a control group without the addition of a biological filler. In addition, the component of the biological filter medium was derived from the land plant *M. polymorpha*. Whole *M. polymorpha* was collected from the edge of an artificial lake at Hangzhou Normal University (N30°19′0.79″, E120°23′56.57″). Deionized water was used to remove dirt that remained on the *M. polymorpha*, which was dried to a constant weight at 60 °C.

To explore the nitrogen and algae removal performance of the BAFs after the addition of the biological filler, the reactors were operated for 50 days without accident. Reactor operation was divided into two stages according to the hydraulic retention time (HRT) and gas-to-liquid ratio: the domestication stage (phase I: HRT = 8 h, gas-to-liquid ratio = 10:1, 1–30 days) and the stabilization stage (phase II: HRT = 4 h, gas-to-liquid ratio = 4:1, 31–50 days). The dissolved oxygen (DO) was maintained between 4.0 and 5.0 by regulating the air flow rate.

### 2.3. Collection and Characterization of Aquaculture Wastewater

The influents and effluents from BAFs were collected for regular testing. Water samples were tested daily for NH_4_^+^-N, NO_2_^−^-N, and NO_3_^−^-N using conventional methods [18]. Excitation–emission matrix (EEM) fluorescence spectra of the influent and effluent were obtained via a fluorescence spectrophotometer (F-4600, Hitachi Co. Ltd., Hitachi, Japan). The TOC concentration was acquired using a TOC analyzer (TOC-L, Shimadzu, Kyoto, Japan). The cell density and concentration of chlorophyll-a (Chl-a) are common indicators for expressing the growth of algal cells. The density of algal cells in aquaculture wastewater was measured via the hemocytometer method [19]. Chlorophyll a (Chl-a) content was used as an indicator of the potential photosynthetic capacity of *M. aeruginosa*, whose concentration affects the growth and photosynthesis of algal cells [20]. Chl-a was extracted with 95% ethanol at 4 °C for 24 h [21]. In brief, 10 mL of each group was collected according to the previous sampling schedule and centrifuged for 10 min at 8000 r/min to obtain a clear supernatant. Then, the absorbance of the supernatant was determined at 665 nm and 649 nm in a spectrometer. The Chl-a content (mg/L) was calculated by Equation (1):

Chl-a = 13.95 × OD_650_ − 6.88 × OD_649_ (1)

where Chl-a denotes the concentration of chlorophyll-a, while OD_649_ and OD_650_ denote the absorbance at 649 and 650 nm, respectively. 

Additionally, the maximum photochemical quantum yield (Fv/Fm), relative electron transport rate (rETRmax), and photosynthesis efficiency (initial slope, alpha) in the aquaculture wastewater were measured with a phytoplankton analyzer (WALZ, Rohrdorf, Germany), according to Wang et al. [22] All samples were exposed to a dark environment for 10 min before measurement. All physiological indicators were measured in triplicate and reported as the mean standard deviation. 

### 2.4. Microbial Community Analysis

As previously reported, high-throughput sequencing of 16S rRNA genes was conducted for to characterize the microbial communities of the BAFs [23]. In brief, the bacterial primers 515F and 907R were used to amplify the V3-V4 region of the 16S rRNA genes [24]. Personal Biotechnology Co., Ltd. (Shanghai, China) constructed libraries and sequenced amplicons from samples. The sequencing findings were analyzed using QIIME software, which included quality filtering and taxonomy classification [25]. A value of 97% was classified as the threshold for describing the operational taxonomic units (OTUs).

## 3. Results and Discussion

### 3.1. Nitrogen Removal Performance of BAFs 

**Figure 2:**
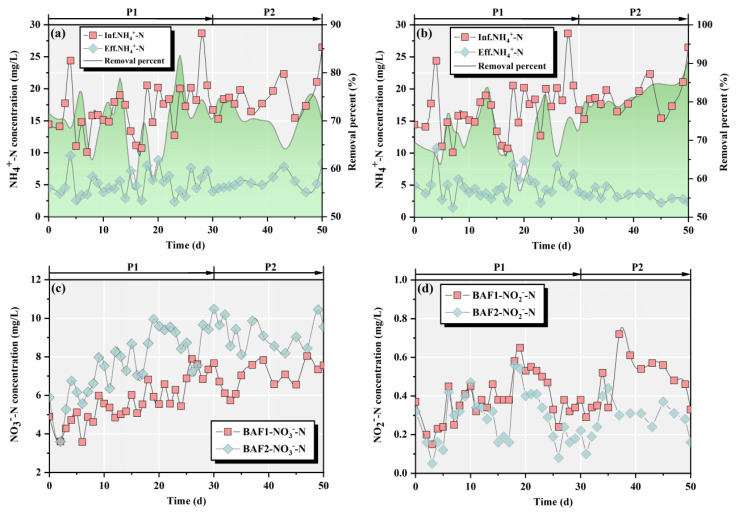
NH_4_^+^-N removal performance of BAF1 (**a**) and BAF2 (**b**) during the whole period; the effluent concentrations of NO_3_^−^-N (**c**) and NO_2_^−^-N (**d**) for BAF1 and BAF2.

The NH_4_^+^-N removal performances of the two BAFs are shown in Figure 2a,b. Over 50 days, BAF1 and BAF2 operated quite well, with average NH_4_^+^-N removal percentages of 70.2% and 74.4%, respectively. Nevertheless, the concentrations of NH_4_^+^-N in the BAF effluents exhibited different trends during the whole period. After the domestication stage, changing the water/gas ratio and HRT in BAF2 had no significant negative impact on the NH_4_^+^-N removal performance. In contrast, the NH_4_^+^-N removal performance of BAF2 was effectively improved in phase II, and the average NH_4_^+^-N removal percent was increased to 82.1%. The average NH_4_^+^-N removal percent of BAF1 was 71.5% in phase II. Compared to BAF1, BAF2, with the biological filler medium, demonstrated better NH_4_^+^-N removal performance. As displayed in Figure 2c, the effluents of the two BAFs had comparatively low NO_3_^−^-N concentrations. The average concentrations of NO_3_^−^-N in the BAF1 and BAF2 effluents were 6.07 and 8.17 mg/L, respectively. In addition, the average concentrations of NO_3_^−^-N in BAF1 and BAF2 remained at low levels during the whole period at 0.41 and 0.28 mg/L, respectively. Based on these findings, the ammonium in the influent could easily be converted into nitrate throughout the BAF system, and there was no large accumulation of nitrite. This result confirmed that the addition of *M. polymorpha* did not negatively affect the denitrification performance of the BAF.

### 3.2. Algae Removal Efficiency of BAFs

**Figure 3:**
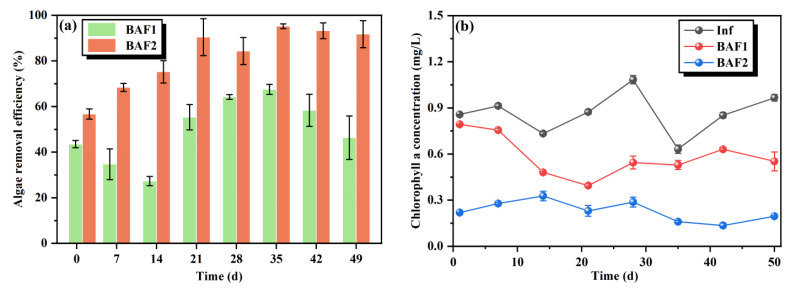
Algae cell removal efficiencies (**a**) and chlorophyll a concentrations (**b**) of BAF1 and BAF2 during the whole period.

The massive presence of ammonia nitrogen in natural water can generate harmful blooms [11]. Therefore, it is important to investigate the algae removal efficiency of the BAF with *M. polymorpha* as a filler medium. As shown in Figure 3a, the algae removal efficiencies of BAF1 and BAF2 demonstrated some fluctuations during the operation of the reactors. The average algae removal efficiencies of BAF1 and BAF2 were 49.6% and 81.9%, respectively. Previous studies have shown that a composite BAF packed with a suspended biological carrier, zeolite, and granular activated carbon had an average algae removal efficiency of 78.6% [26]. Compared to the composite BAF, the addition of *M. polymorpha* as a filler medium into the BAF was more beneficial in enhancing the removal of algal cells. Furthermore, BAF1 could reduce the effluent algal cell density, which may be attributed to the presence of the two filter media in BAF1: zeolite and activated carbon. Zeolite and activated carbon have large specific surface areas and microporous structures which can remove algae by adsorption [27]. However, the algae removal efficiency of BAF1 on days 42 and 49 was significantly lower compared to that on Day 35. Presumably, with the long-term operation of BAFs, massive algal cells adsorb on the surface of the filter media, which increases the clogging risk of the reactor and thereby decreases the algae removal capacity. In addition, the higher algae removal efficiency of BAF2 may benefit from the addition of *M. polymorpha*. On one hand, the algal cells of the influent were first intercepted by *M. polymorpha*, which reduced the algal cell density of the effluent. On the other hand, *M. polymorpha* contained a variety of sesquiterpenes, which can effectively inhibit the growth of algae cells. Chlorophyll-a (chl-a) concentration was used as an indicator of the potential photosynthetic capacity of algal cells. As shown in Figure 3b, after 50 days, the average chl-a concentrations of the effluents in BAF1 and BAF2 were 0.585 mg/L and 0.229 mg/L, respectively, which were much lower than that of the influents (0.864 mg/L). This result was consistent with the algae removal efficiencies of BAF1 and BAF2, suggesting that the addition of the *M. polymorpha* filler enhanced the algae removal ability of the BAF system. 

**Figure 4:**
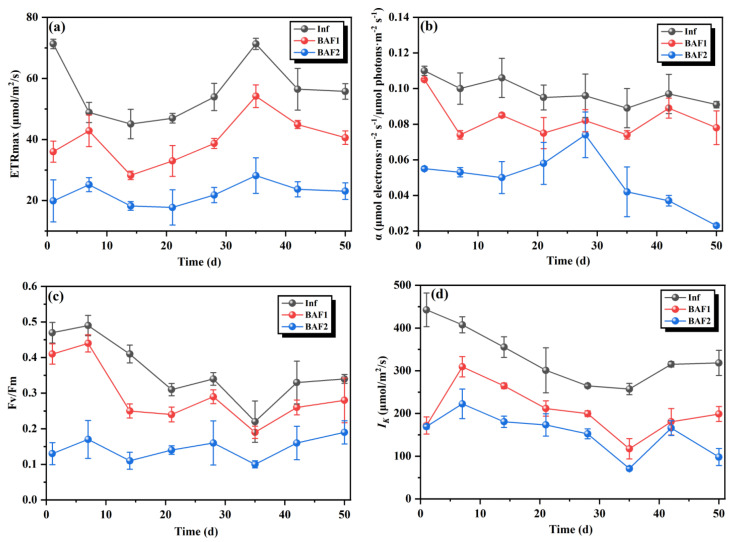
Performances of BAF1 and BAF2 in the aquaculture wastewater degradation experiment based on the maximal electron transport rates (rETRmax) (**a**), α (**b**), maximum efficiency of photosystem II (Fv/Fm) (**c**), and semi−light saturation point (Ik) (**d**) during the whole period.

To more comprehensively and systematically study the algae removal mechanism of the BAF system with the *M. polymorpha* filler, we further measured the photosynthetic activity parameters of the influent and effluent. As shown in Figure 4a,b, the rETRmax and α values in the effluent were noticeably lower than those in the effluent after the BAF1 treatment (without the *M. polymorpha* filler) and BAF2. The rETRmax and α values indicate the photoacclimation state of the algae, which is often used as a response to the energy transfer and capacity of the electron transport chain in algal cells [28]. Compared to BAF1, the rETRmax and α values of the BAF2 effluent decreased to a greater extent, and the average values were 22.221 and 0.049, respectively. This result suggests that the electron transport chain transmission of the algal cells was inhibited by the BAF system with the *M. polymorpha* filler. The Fv/Fm and Ik values of the effluent were also altered by the BAF system (Figure 4c,d). Fv/Fm and Ik are important indicators of the light absorbed by PSII. When the Fv/Fm value is below 0.3, this indicates poor algal health [29]. The average Fv/Fm values for the BAF1 and BAF2 effluents were 0.295 and 0.145, respectively. At the same time, the Ik value of the BAF2 effluent was significantly lower than that of the influent. These results indicate that the BAF system with the *M. polymorpha* filler caused more severe damage to the PSII of the algal cells. 

Algae is an autotrophic species that relies on its photosynthetic system for energy conversion [30]. The *M. polymorpha* filler medium severely interfered with the capture and conversion of light energy by the algal cells in the aquaculture wastewater, leading to a complete collapse of the photosynthetic system. Therefore, biological aerated filters with *M. polymorpha* as the filler medium have potential development prospects for application in aquaculture wastewater treatment.

### 3.3. TOC Removal Performance of the BAFs

**Figure 5:**
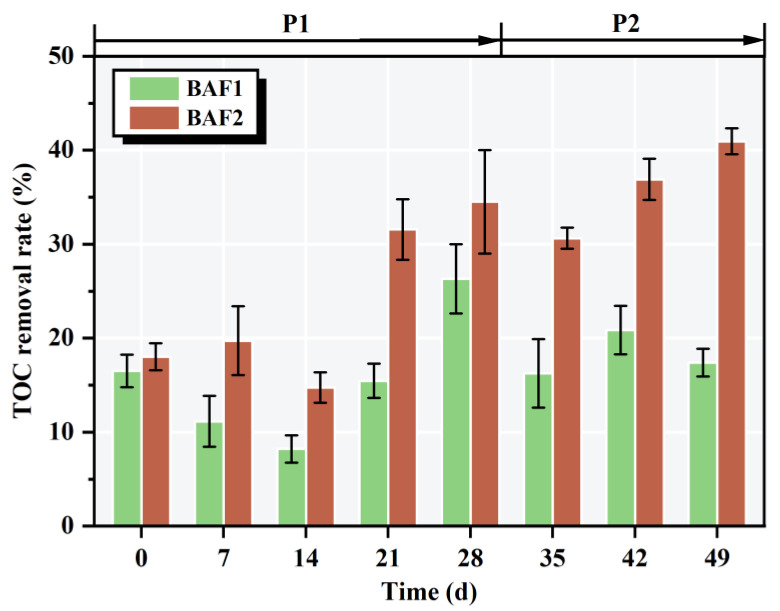
Performances of BAF1 and BAF2 in the aquaculture wastewater degradation experiment based on TOC concentration during the whole period.

As shown in Figure 5, there was a significant difference in the TOC removal performance between BAF1 and BAF2 during the whole operation. The reduction in TOC may be attributed to the microbial activity in the sludge. On one hand, microorganisms consume large amounts of carbon for their own growth and metabolism [31]. On the other hand, the microorganisms in the sludge also need to obtain carbon from the wastewater for the energy to carry out denitrification [32]. In the domestication stage (P1), the average TOC removal rates in the reactors were 15.53% (BAF1) and 23.71% (BAF2). In general, it takes a long time to completely adapt the microbial community of the inoculum to the BAF system. At this stage, the microbes were unbound and readily oxidized, resulting in limited microbial activity [11]. Therefore, the TOC removal rates of BAF1 and BAF2 fluctuated greatly in P1, and the average removal rate was poor. However, in phase II, the TOC removal rate of BAF2 gradually increased, and the average removal rate of TOC in BAF2 increased to 37.49%. This result may be attributed to the microbial activity in the second stage of BAF2 being better than in the first stage, and excessive organic matter in the algae-containing wastewater being metabolized by heterotrophic microorganisms [33]. Furthermore, the average TOC removal rates in the BAFs were 16.52% (BAF1) and 28.38% (BAF2) during the whole operation, indicating that BAF2 exhibited the best performance in TOC removal. It is reasonable to assume that the addition of the *M. polymorpha* filler medium could enhance the community structure of the functional microbes in the BAF system.

### 3.4. EEM Fluorescence Spectral Analysis

**Figure 6:**
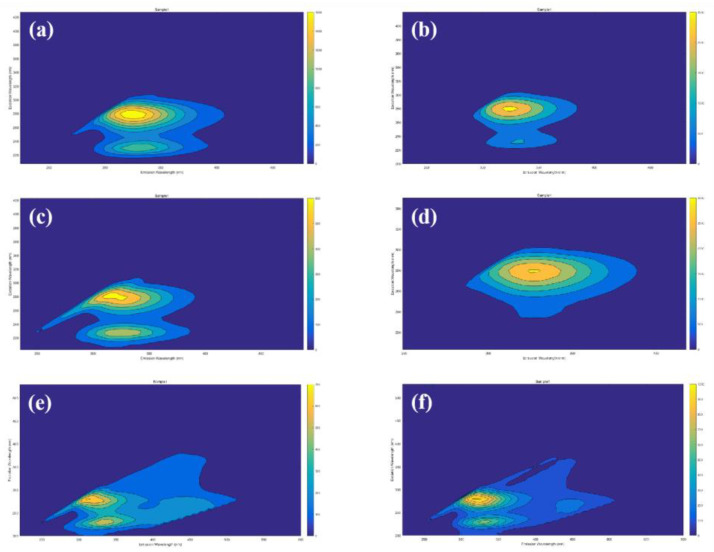
EEM fluorescence spectra of influents and effluents from the different BAFs during the experiments; (**a**,**b**)—EEM fluorescence spectra for influents in phases I and II; (**c**,**d**)—EEM fluorescence spectra for effluents from BAF1 and BAF2, respectively, in phase I; (**e**,**f**)—EEM fluorescence spectra for effluents from BAF1 and BAF2, respectively, in phase II.

In this study, changes in the type and content of organic matter in the aquaculture wastewater were detected via EEM fluorescence spectra during phase I and II. As shown in Figure 6, two peaks can be identified in the EEM spectra. The first peak represented aromatic proteins (Ex/Em: 220–250/330–360 nm) [34]. The second peak represented soluble microbial products (SMPs) (Ex/Em: 250–300/330–360 nm) [34]. Aquaculture wastewater has a massive amount of algal cells, and the algal cells release numerous extracellular compounds to the external environment; these compounds are mainly protein-like substances [35]. Therefore, the peak with the highest fluorescence intensity in the EEM fluorescence spectra of influents was identified as belonging to a protein-like substance (Figure 6a,b). The fluorescence intensity of the protein-like substance (PLS) in the effluent water decreased significantly after BAF treatment. The fluorescence intensity of the protein-like substance was higher in BAF1 than in BAF2 (Figure 6c,e). In BAF2, the *M. polymorpha* filler medium decreased the algal cell density in the influent, which limited the release of extracellular substances. Hence, the PLS fluorescence intensity in the BAF2 effluent was lower than that in the BAF1 effluent. This result was also consistent with the algae removal performance of BAF2. Additionally, in the phases I and II, the SMP peak fluorescence intensity of the BAF2 effluent was higher than that of the BAF1 effluent. In BAF2, the *M. polymorpha* filler medium provided an additional carbon source for microorganisms; thus, the SMP peak fluorescence of the BAF2 effluent was higher than that of the BAF1 effluent. Moreover, microorganisms consume carbon sources to maintain their metabolic activities and denitrification, which will also lead to an increase in the fluorescence intensity of SMPs [23]. According to the results of 3.1 and 3.3, the addition of the *M. polymorpha* filler medium improved the performance of the removal of TOC and NH_4_^+^-N from BAF2. Simultaneously, the microorganisms in BAF2 were adapted to the environment with the *M. polymorpha* filler medium, so a higher SMP fluorescence intensity from the BAF2 effluent was detected.

### 3.5. Microbial Community Analysis

The microbial community structures of the biofilms attached to the *M. polymorpha* filler medium from BAF1 and BAF2 was measured via high-throughput sequencing. As shown in Table 2, 44,677, 45,474, and 51,531 effective sequences were attained for the microbial samples on the initial sludge (BAF0), BAF1, and BAF2, respectively. The Chao 1 and ACE indexes of BAF2 were higher than those of BAF1, which indicated that the microbial community richness for BAF2 was greater than for those attached to BAF1. Furthermore, the Simpson and Shannon indexes for BAF2 were also significantly higher than those of BAF0, suggesting that the biofilm in BAF2 had a high level of microbial diversity. Based on the above results, the addition of the *M. polymorpha* filler medium was conducive to microbial enrichment and improved the richness and diversity of the microbial community.

**Figure 7:**
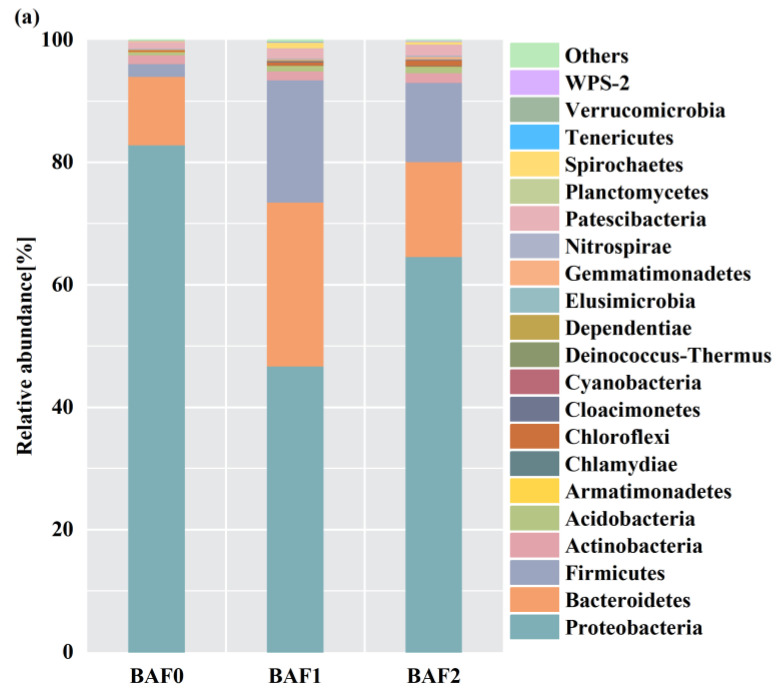

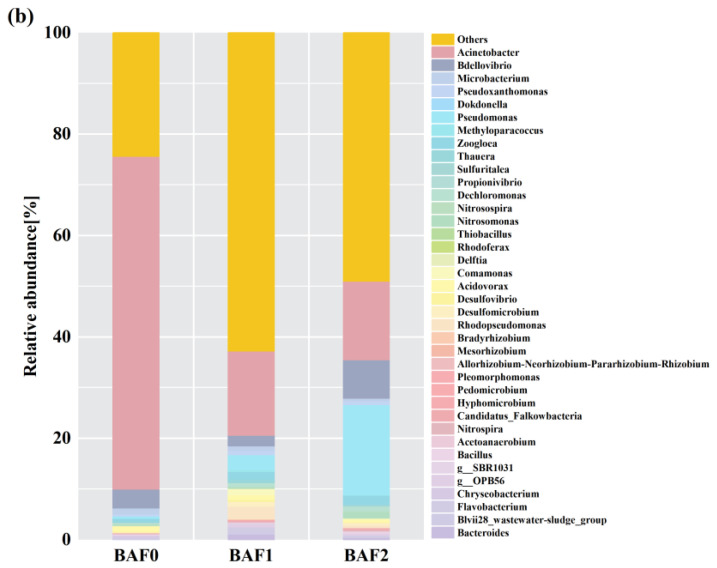
Microbial population dynamics of the initial sludge (BAF0), BAF1, and BAF2 microbial samples at the phylum (**a**) and genus (**b**) levels.

As shown in Figure 7a, *Proteobacteria* was the most dominant phylum in all the microbial samples from BAF0, BAF1, and BAF2, accounting for 82.8%, 46.7%, and 64.6% of the total effective reads, respectively. Compared with BAF1, the abundance of *Proteobacteria* and *Bacteroidetes* was higher in BAF2. *Proteobacteria* have been widely detected in drinking water, wastewater, soils, and sludge [36]. In addition, bacteria belonging to *Proteobacteria* can remove nitrogen in anaerobic, aerobic, and anoxic environments [37]. A higher abundance of such bacteria would be conducive to improving the nitrogen removal performance of BAF. *Bacteroidetes* are essential for denitrification because they participate in the anaerobic or anoxic hydrolysis of macromolecular molecules [38]. The nitrogen removal performances of the two reactors differed owing to differences in the relative abundances of *Bacteroidetes*. Moreover, *Firmicutes* was also one of the most dominant phyla in the two reactors. The significance of *Firmicutes* in the denitrification process, utilizing solid carbon sources, has been widely mentioned [39]. *Acidobacteria* showed higher abundances in BAF1 and BAF2 than BAF0, which was ascribed to the addition of activated carbon and zeolite in the BAF system.

To obtain a better comprehension of the bacterial structure, the bacterial abundances of BAF0, BAF1, and BAF2 at the genus level were investigated. As shown in Figure 7b, at the genus level, *Actinobacteria* was the most dominant genus in BAF1 and BAF2, accounting for 16.6% and 15.5%, respectively. *Actinobacteria* is a genus of sheathed filamentous bacteria that has been discovered in several wastewater treatment systems [40]. After 50 days of reactor operation, *Bdellovibrio* and *Pseudomonas* had notably higher abundances in BAF2, increasing significantly and accounting for 7.6% and 17.8%, respectively. Previous research has shown that *Bdellovibrio* plays an essential role in NO_3_^−^-N removal under denitrifying circumstances [41]. *Pseudomonas*, an aerobic denitrifying bacterium, has an excellent ability to convert nitrate into nitrogenous gases [42]. 

Overall, the above results indicate that BAF2, with *M. polymorpha* filler medium, was the most microbiologically active reactor. Meanwhile, *M. polymorpha* filler medium enriched the dominant phylum that was capable of nitrification and correspondingly played a major role in the denitrification of aquaculture wastewater. Hence, the *M. polymorpha* filler medium exhibits promise in aquaculture wastewater treatment which is worth investigating in the future.

## 4. Conclusions

A biological aerated filter equipped with *M. polymorpha* filler medium was proposed for the first time in this research, which contributed to establishing an efficient and reliable pretreatment system for aquaculture wastewater. In this work, the use of the low-cost and easily available terrestrial plant, *M. polymorpha,* as a filler medium greatly enhanced the removal of ammonium ions (NH_4_^+^) and algal cells from aquaculture wastewater. The main contribution of *M. polymorpha* was the removal of algae cells from aquaculture wastewater by interfering with the photosynthetic system of the algae cells. On the other hand, the *M. polymorpha* filler medium could enrich denitrifying microorganisms such as *Bdellovibrio* and *Pseudomonas* and improve the denitrification performance of the BAF. This study provides vital insights into aquaculture wastewater pretreatment and a theoretical basis for the development of BAFs with *M. polymorpha* filler media in the future.

## Figures and Tables

**Table 1:** Characteristics of BAF system influents.

NH_4_^+^-N (mg/L)	NO_2_^−^-N (mg/L)	NO_3_^−^-N(mg/L)	Algae Density (10^8^ Cell/L)
15 ± 0.58	0	5 ± 0.42	1.65 ± 0.64

**Table 2:** The richness and evenness indices of BAF0, BAF1, and BAF2 microbial communities.

Sample	Sequence	Chao1	ACE	Simpson	Shannon
BAF0	44,677	446.0	412.9	0.9510	5.7765
BAF1	45,474	744.3	689.2	0.9841	7.4083
BAF2	51,531	835.3	753.6	0.9716	7.0655

## Data Availability

Not applicable.
